# Microbiological features distinguishing Lyme disease and relapsing fever spirochetes

**DOI:** 10.1007/s00508-018-1368-2

**Published:** 2018-08-03

**Authors:** Sven Bergström, Johan Normark

**Affiliations:** 1 Department of Molecular Biology, Umeå University, 6K och 6L, Sjukhusområdet, 901 87 Umeå, Sweden; 2 Department of Clinical Microbiology, Umeå University, Umeå, Sweden; 3 Umeå Center for Microbial Research, Umeå University, Umeå, Sweden; 4 Molecular Infection Medicine Sweden, Umeå University, Umeå, Sweden

**Keywords:** Borrelia, Borreliella, Lyme borreliosis, Relapsing fever, Taxonomy

## Abstract

The recent proposal of splitting the genus *Borrelia *into two genera in the newly formed family of *Borreliaceae*, i. e. *Borrelia* and *Borreliella* has motivated us to reflect upon how these organisms has been characterized and differentiated. This article therefore aims to take a closer look on the biology and virulence attributes of the two suggested genera, i. e. those causing Lyme borreliosis and relapsing fever borreliosis. Both genera have much in common with similar infection biological features. They are both characterized as bacterial zoonoses, transmitted by hematophagous arthropods with almost identical microbiological appearance. Nevertheless, a closer look at the genotypic and phenotypic characteristics clearly reveals several differences that might motivate the suggested split. On the other hand, a change of this well-established classification within the genus *Borrelia* might impose an economical burden as well as a great confusion in society, including medical and scientific societies as well as the general population.

## Introduction

Recent molecular analysis of *Borrelia *spirochetes has suggested a new taxonomic classification. Thus, spirochetes within the family *Borreliaceae *[1], which belongs to the order Spirochetales, has recently been reclassified and now been suggested to contain two different genera; *Borrelia*, and* Borreliella *[2]. The genealogical separation of relapsing fever and Lyme disease spirochetes has been debated and questioned among researchers, clinicians and stake holders within the borreliosis community [3, 4]. The suggested split is based on an in-depth molecular analysis of phylogenetic and phenotypic characteristics, i. e. genome and proteome comparison of the increasingly available omics data. The results from the comparative analyses partly support this separation, which will be further discussed in this review.

Although relapsing fever (RF) was described during antiquity it acquired its current name much later. About 430 BC Hippocrates described an epidemic fever that was probably related to RF. Several outbreaks of an “epidemic fever” during the following centuries have been attributed to RF mainly because of the disease symptoms [5]. The first description of clinical features associated to RF was documented in the middle of the eighteenth century from an outbreak in Ireland. This epidemic spread all over the British Isles and the disease was for the first time labelled “relapsing fever” in 1843 [5, 6]. The disease was then further disseminated to the European continent and the USA. The outbreaks continued for decades and plagued large parts of the world [5]: however, it is important to remember that at this time other microorganisms could have been the etiologic agent for this recurrent fever, including *Rickettsiae*, malarial parasites or various viruses. None of these historical descriptions are definite but are of course interesting from an epidemiological point of view. The suspected RF infection was most often found in poor and overpopulated areas, and therefore the body louse was assumed to spread the disease. The importance of the body louse, *Pediculus humanus humanus* in transmitting RF was later shown experimentally by inoculating monkeys with crushed lice from human RF patients [6]. In 1868 the RF microorganism was discovered and described by the German doctor Otto Obermeier and initially designated “*Spirocheta Obermeieri*,” but finally denoted its current name *Borrelia recurrentis *[5].

Lyme disease is of much later date, i. e. the date of when the disease was defined and its etiologic agent was proven and described. The Lyme borreliosis era started when several cases of arthritis were reported and described in 1977 in the town of Old Lyme, Connecticut, USA. This came from an epidemic of oligoarthritis in children, which first was misdiagnosed as being juvenile arthritis [7]. Interestingly, the patients often developed an expanding skin rash prior to onset of arthritis. Therefore, these findings led to the suspicion that the diagnosis was incorrect. This pathognomonic skin rash, termed erythema migrans, was first reported by a Swedish dermatologist Arvid Afzelius in the year 1909 [8]. He suggested that a tick bite caused this particular type of skin lesion. Similar to the finding by Afzelius, Steere et al. also believed that ticks were involved in the transmission of this novel disease [9]. Therefore, it was proposed that the symptoms resulted from transmission of an infectious agent by an arthropod vector. Subsequently, a spirally formed bacteria could be isolated from the American Deer tick, *Ixodes scapularis* and a similar organism was later isolated from the blood and cerebrospinal fluid from patients with erythema migrans [9–12]. Further microbiological and biochemical characterization of this microorganism suggested that the spirochete-like organism belonged to the genus *Borrelia*. Thus, these investigators now suggested this novel microorganism to be a *Borrelia *spirochete because of its microbiological and microscopically resemblance to RF *Borrelia. *This *Borrelia-*like spirochete was then named *Borrelia burgdorferi *after one of its co-discoverers, Willy Burgdorfer [12].

## Basic characteristics

The typical symptom of Lyme borreliosis is the characteristic skin rash denoted erythema migrans. Untreated, the disease can in certain individuals disseminate and cause symptoms in the nervous system or joints as well as the more persistent skin disorder acrodermatitis chronica athrophicans. For a more thorough description of the various clinical symptoms of Lyme borreliosis see Review by Stanek et al. [13]. Nevertheless, Lyme borreliosis is characterized by the deleterious effects on specific tissues, including the skin, central nervous system, joints and the heart. In contrast to the tissue specificity of Lyme disease, relapsing fever (RF) borreliosis initially starts as a blood-borne disease and is characterised by a high concentration of spirochetes in the blood and intermittent attacks of high fever. In essence, the main disease manifestation is a recurring fever that coincides with high numbers of spirochetes in the blood. The severity of the infection ranges from asymptomatic to serious.

Despite the suggestion to separate the spirochetes of the new family *Borreliaceae* into two genera, they still have several general microbiological features in common. They are characteristically spiral shaped, approximately 25–30 µm long and 1 µm thick [14]. A second typical feature of the organisms is the locomotor organ that contains a bundle of flagellae attached to the poles. The flagellae are located in the periplasmic space and wrapped around the cell cylinder, giving it its characteristic shape [14–16]. The outer membrane is Gram-negative, meaning that it has a diderm structure that contains a cytoplasmic-and an outer membrane separated by the periplasm [17, 18]. The spirochaetal outer membrane is regarded as more fluid than other Gram-negative bacteria with a 45–62% protein, 23–50% lipid and 3–4% carbohydrate content [14]. Another important difference compared to other Gram-negative bacteria is the absence of lipopolysaccharides on the outer surface. Instead, *Borrelia *spirochetes have a large amount of surface located lipoproteins and as much as ~8% of the open reading frames in *Borreliae* encodes for lipoproteins [14, 18, 19]. Many of the surface located lipoproteins are thought to be important for the life style of the *Borreliae* and therefore important factor(s) during infection.

## Virulence factors

### Lyme borreliosis

Several factors important for the pathogenesis of the various species within the family of *Borreliaceae *has been described and characterised both in vitro and in vivo. Virulence factors are defined as any specialized virulence trait that separates an infectious bacteria from a non-virulent variant. Typically, those factors are involved in adhesion, colonization, invasion and toxin production. Several virulence factors important for Lyme borreliosis has been described and investigated during the last few decades. The investigation of Lyme borreliosis virulence traits took off with the publication of the *B. burgdorferi* genome by Fraser et al. [18], and with the subsequent full genomic annotation in 2000 [20]. Many unique virulence factors that operate during Lyme borreliosis have been characterized and defined. These factors include various outer surface molecules that interact with the mammalian host. Among these are several cell surface outer membrane proteins (OMPs), which can act as adhesins or receptors for different molecules and aid in attachment, transmission and immunological escape of the pathogen. It is furthermore well known that *Borrelia spp*. express different parts of the OMP repertoire depending on if it resides in a mammalian or a tick host.

Several important adhesins have been characterized that are essential during Lyme borreliosis. Among those are the DbpA and DbpB surface located proteins, which bind to the proteoglycan decorin [21–23]. Additionally, expression of DbpA and DbpB was shown to be up-regulated at 35 °C versus 23 °C and it was further shown that the presence of antibodies against DbpA could prevent a *B. burgdorferi *infection indicating a possible use as a vaccine. Disappointingly, in vivo challenge studies showed that the efficacy was not as pronounced as for other Lyme borreliosis vaccine candidates, e.g. OspA [23–25]. In conclusion, the results suggest that certain adhesive properties by these decorin-binding proteins are important for the colonization of the human host.

Another characterized adhesin is BBK32 a fibronectin-binding protein that promotes *B. burgdorferi *attachment to glycosaminoglycans (GAG) [26]. Similarly to DbpA and DbpB, the expression of BBK32 is also up-regulated at 35 °C versus 23 °C indicating its importance in the mammalian host. Likewise, an inactivation of BBK32 leads to a significantly attenuated infectious phenotype of *B. burgdorferi*, indicating that the fibronectin-BBK32 interaction might be important for *B. burgdorferi *pathogenesis [25, 27].

**Fig. 1:**
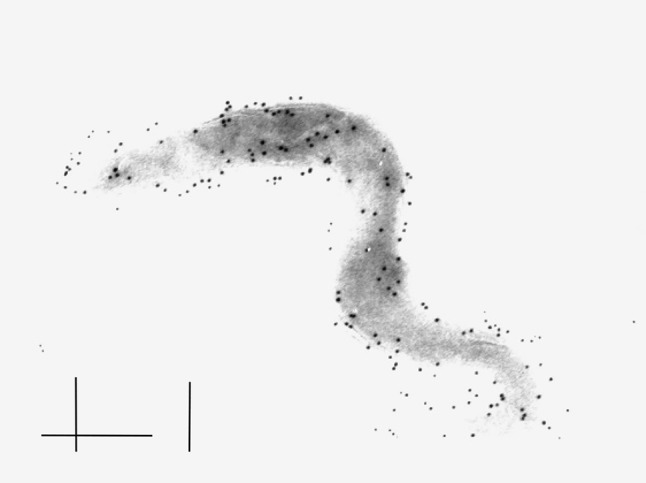
Immunogold labelled P66 porin of *B. burgdorferi* strain B31 using monospecific polyclonal rabbit antiserum. The bar corresponds to 0.5µm. Photo: Lenore Johansson. Reproduced with permission from Oxford Academic Press

Of all the different adhesins described in *Borrelia *spirochetes, perhaps P66 is one of the most interesting, in part because of its dual function. Hence, P66 has been shown to both act as a porin and as a mediator of interaction between *B. burgdorferi *and beta_3_-chain integrins, molecules that are found on various immune cells, blood platelets and endothelial cells. Although the function of P66 is not elucidated the integrin-binding activity of P66 is thought to aid in *Borrelia *escape from the site of the inoculum and in dissemination of the bacteria into tissues [28, 29]. The P66 adhesin is also the most studied porin in *Borrelia *and has a remarkably high single channel conductance indicating a possible unique role for these spirochetes ([30–32]; Fig. 1).

Also, many factors important for the dissemination of *Borrelia *spirochetes either hematogenously or directly through various tissues have been characterized. Most important in this process is the chemotaxis and motility apparatus that enables the spirochete to efficiently spread to various sites in the mammalian host [15]. An in-depth description of the molecular function of the motility and chemotaxis and its role during Lyme borreliosis has been summarized in [15, 33].

A functionally diverse and large group of virulence factors are the complement regulatory acquiring surface proteins (CRASPs). These proteins can bind complement inhibiting factor H (CFH), factor H-like protein 1 (CFHL-1) and several other factor H-related proteins (CFHRs). When binding to these complement-inhibiting proteins, the *Borreliae* can evade host-mediated complement killing [34–38]. Until now, several CRASPs have been found in *B*. *burgdorferi, *including CspA, CspZ, ErpA, ErpC and ErpP.

The most prominent surface proteins of Lyme borreliosis *Borrelia* are the major Osps, i. e. OspA, OspB and OspC. These major Osps, exhibit a variable expression with high expression level of OspA and OspB in the tick and a simultaneous shift to OspC expression during feeding and after entering the mammalian host [39]. Thus, it appears that OspC expression is coupled to the spirochete transmission, as well as the presence within the mammalian host. Likewise, the OspA/B expression is associated with the arthropod host [39, 40]. Both OspA and OspC have adhesive properties that aid in the transmission of the spirochete from tick to mammal.

Another interesting surface located lipoprotein is the *vlsE*, which is a 35 kDa lipoprotein of Lyme borreliosis *Borrelia *that is analogous to the VMP’s of RF *Borreliae* (see below) and can undergo antigenic variation. This antigenic variation mechanism is used by the bacteria to escape recognition and elimination by the host immune system. The expression site of *vlsE *together with a continuous site of 15 *vls* silent cassettes comprise the *vls *locus [41, 42]. The plasmid carrying the *vlsE*, lp28-1, is highly correlated with an infectious phenotype of *B. burgdorferi*, which is believed to be mainly due to the *vls *locus [43, 44].

### Relapsing fever borreliosis

Many of the virulence factors described for Lyme borreliosis borreliae also occur in RF spirochetes, although not so thoroughly investigated. The infectious course during RF borreliosis, i. e. from the initial deposition of the spirochete in the skin to the subsequent colonization in various tissues, exposes the spirochetes to many defense mechanisms of the host. During the course of infection it occupies a variety of different environments or niches, including the skin, the circulation, and eventually peripheral organs with their own specific milieu. It means that the RF *Borrelia *will combat several components of the host immune system. To accomplish survival in all different environments the spirochete has developed mechanisms to adapt and survive in different surroundings of the animal host, which then defines its virulence characters.

An RF borreliosis infection initially starts with either a tick bite or by a louse blood meal. Usually, the tick bite is painless and therefore unnoticed. The anesthesia is caused by the inoculation of a variety of effectors secreted along with the tick saliva, including pain killers, anticoagulation factors and anti-inflammatory substances. All these factors are important for a successful infection and support the tick in obtaining a successful blood meal with a subsequent transmission of the disease [45–47]. In addition, tick saliva inhibits the activity of polymorphonuclear leucocyte (PMN), which further reduces the killing of the spirochete [48]. Next, the RF *Borrelia* must penetrate both the extracellular matrix (ECM) and the endothelial lining of the blood vessels in order to maintain and spread the infection to adjacent tissues. As for Lyme borreliosis spirochetes the characteristic shape as well as the motility and chemotaxis mechanisms are important for the dissemination, as demonstrated by the loss of endothelial penetration ability of flagella mutants [49]. Also, the host protease activity is part of the spreading process as shown by studies in plasminogen-deficient mice (*plg*^*−/−*^), which demonstrated that dissemination of RF *Borrelia* to tissues is delayed when the plasminogen activator system (PAS) is missing, and with a simultaneous reduction of bacterial load in the brain and the heart of infected animals [50, 51]. Additionally, an activation of matrix metalloproteinases (MMP’s) also follows plasminogen binding and activation on the spirochete surface [52]. Thus, the conclusion is that utilization of host proteases supports spreading of the RF *Borrelia *spirochete.

**Fig. 2:**
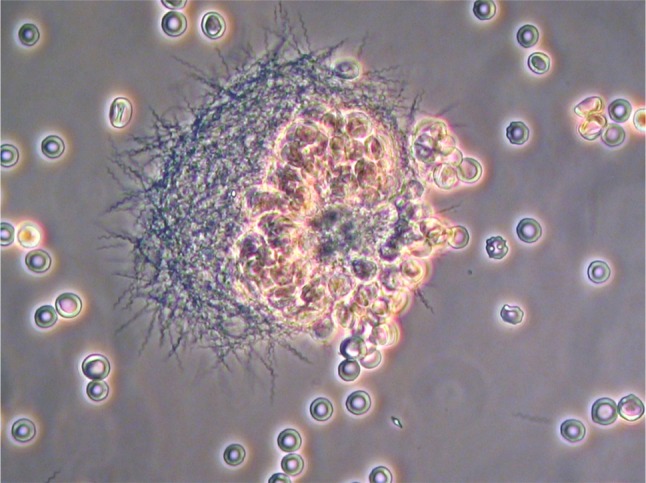
Interaction between erythrocytes and the relapsing fever species *B.* *duttonii* (photo: Marie Andersson)

As mentioned, RF borreliosis is in the initial stages a blood-borne infection that subsequently multiplies in the circulation, where it reaches very high numbers. Several of the Old World RF *Borrelia* species, e. g. *B. duttonii, B. crocidurae*, and *B. hispanica*, frequently interact with cells in the blood, primarily the erythrocytes. Thus, these Old World RF species cause the red blood cells to aggregate, a phenomenon termed erythrocyte rosetting (Fig. 2). The mechanism of red blood cell rosetting was first shown by Mooser in 1958 [53]. Moreover, in vitro models later suggested that the interaction is a way for the spirochete to cover itself with components that may inhibit detection by the immune response [54]. Another hypothesis, although not proven, is that the spirochete forages on the erythrocytes, i. e. collecting nutrients from the cells during its interaction with erythrocytes. Throughout an infection with RF *Borrelia *there are large amounts of spirochetes present, reaching up to 1 billion of spirochetes per milliliter of blood. Thus, it is evident that a considerable amount of nutrients are needed to support the growth of the RF borreliae population in the blood. The grazing theory was further backed-up by the findings that RF *Borrelia* species have specific genes that enable them to use purines from serum as metabolites for synthesis of various macromolecules [55]. These particular genes are not present in Lyme borreliosis spirochetes that interestingly never reach high densities in the blood displaying an important difference between these two genera. In addition, the purine hypoxhantine is abundant in human plasma and also produced by erythrocytes indicating a possible interaction with red blood cells as a means for nutrient acquisition as well as other metabolites needed for growth [55]. The RF *Borrelia *interactions with cells in the circulation might be a virulence strategy to increase and lengthen the time this pathogen can be retained within the host. Besides, erythrocytes and possibly other blood cells can provide nutrients or shelter from the host immune response and might augment penetration into distant tissues and organs.

Next and the most noticeable virulence factor of RF *Borrelia *is the mechanism of antigenic variation. This is the mechanism that drives the antigenic variation of the surface located outer surface variable membrane proteins (Vmp’s) and as such prolongs the infection as long as possible. Numerous reviews and book chapters have been written about this process and are not discussed in detail. In short, the mechanism of RF was described by early researchers as a process in which every relapse of the infection was caused by a newly appearing variant of the original infecting strain. This indicated that the recurrent variant was a result of an antigenic variation of a dominant and immunogenic surface protein, the so called variable major proteins (Vmp) [56, 57]. Throughout every fever peak during RF, the majority of the spirochetes express one type of Vmp, eliciting the host to mount an antibody response towards this serotype that subsequently results in a clearance of this particular serotype from the circulation. In parallel, a second wave of spirochetes with a new serotype will emerge and multiply. These bacteria are expressing a Vmp that is no longer recognized by the immune system and that causes a new bout of spirochetemia with high fever. This process of multiphasic antigenic variation may be repeated several times. Only one Vmp is expressed at one time in a single expression locus, other variants are silent and located on several linear plasmids, but by recombination the expressed Vmp is replaced by one of these silent variants. These bacteria, the new serotype, will start to express the new Vmp and thus evade antibody-mediated killing [57–60].

Finally, the progress of RF involves tissue invasion and colonization connected to an additional escape from the immune defense. A comprehensive and recently published review article by Talagrand-Reboul et al. summarizes the infection biology of RF borreliosis [61].

As summarized in this review the RF and Lyme borreliosis spirochetes have many characteristics in common, including the overall organization of the genome with a well-conserved synteny of the chromosome displayed as a very similar gene order. In contrast, a larger variability is seen in the various linear and circular plasmids of the different spirochetal types, i. e. RF and Lyme borreliosis borreliae. Although both types are transmitted by ticks and most are characterized as bacterial zoonoses there are several prominent differences between the two groups.

The RF group presents a more variable disease spectrum, including tick-borne and louse-borne RF as well as the separate and specific types of avian and bovine borreliosis. In contrast, Lyme borreliosis is more homogeneously characterized by the different stages of disease presented in various tissues. There is also a different disease progression that separates these two types of spirochetes as RF borreliosis is a blood-borne disease with a large amount of spirochetes in the blood whereas Lyme borreliosis is characterized by a very low amount of spirochetes in the blood but a predilection for various tissues, including skin, joints and the nervous system. Moreover, the Lyme borreliosis spirochetes are transmitted by prostriate ixodid ticks while RF spirochetes are more variable and can be transmitted by argasid ticks, prostriate ixodid ticks, metastriate ixodid ticks and human body lice. Another difference is the ability of most RF tick-borne species to undergo transovarial transmission in the ticks.

## Conclusion

This review article does not have the intention to lead the reader to any conclusion or decision about the suggested split of the *Borrelia* genus into two new species, *Borrelia *and *Borreliella. *This suggested split has indeed initiated quite a turmoil within the *Borrelia *community. This article summarizes the basic differences and similarities of these two pathogenic groups of spirochetes, i. e. causing RF and Lyme disease (Lyme borreliosis). In parallel to this split within the genus there is also a recommendation to change the family name of various spirochetes, where *Borrelia *and *Borreliella *will be placed in the new family* Borreliaceae.* In connection to this we also acknowledge the problem of using the collective name *Borrelia burgdorferi *sensu lato for Lyme borreliosis spirochetes as well as the common mix-up between Lyme borreliosis and RF borreliae among lay people and in the healthcare setting. however, there are a number of important and practical caveats regarding the name change. The diagnostics geared to recognize *Borrelia* infections as well as the treatment regimens are anchored in the present nomenclature. It has been painstakingly built by many stakeholders and represents literally billions of dollars in investments from private and public funds. Changing the names would entail added costs with little or no de facto gain. Furthermore, the chances for confusion and misdiagnosis are increased and could in the end result in an increased risk for the patients suffering from the disease. While changes in nomenclature may be pertinent for genetic and taxonomic reasons, any modification has to be well thought through and the risks carefully considered.

We believe that the last word on this issue has not yet been said and we are eagerly waiting for the next step in this *Borreliaceae* drama. Either it might just fade away as happened to the renaming of the genus *Chlamydia chlamydiophila. *Alternatively, a decision similar to the ruling for keeping the name for *Yersinia pestis *instead of giving it the status of being a subspecies of *Y. pseudotuberculosis *as violation of the international code of nomenclature for bacteria [62].
